# Fluvoxamine Exerts Sigma-1R to Rescue Autophagy via Pom121-Mediated Nucleocytoplasmic Transport of TFEB

**DOI:** 10.1007/s12035-023-03885-9

**Published:** 2024-01-05

**Authors:** Chun-Yu Lin, Hsiang-En Wu, Eddie Feng-Ju Weng, Hsuan-Cheng Wu, Tsung-Ping Su, Shao-Ming Wang

**Affiliations:** 1 School of Medicine, China Medical University, Taichung, Taiwan; 2 Neuroscience and Brain Disease Center, China Medical University, Taichung, Taiwan; 3 Cellular Pathobiology Section, Integrative Neuroscience Research Branch, Intramural Research Program, National Institute on Drug Abuse, NIH/DHHS, Suite 3512, 333 Cassell Drive, Baltimore, MD 21224 USA; 4 Graduate Institute of Biomedical Sciences, China Medical University, Taichung, 404333 Taiwan; 5 Department of Neurology, China Medical University Hospital, Taichung, Taiwan

**Keywords:** *C9orf72*-amyotrophic lateral sclerosis, Sigma-1 receptor, Nucleoporin Pom121, Nucleocytoplasmic transport, Fluvoxamine

## Abstract

Expansion of the GGGGCC-RNA repeat is a known cause of amyotrophic lateral sclerosis (ALS) and frontotemporal dementia (FTD), which currently have no cure. Recent studies have indicated the activation of Sigma-1 receptor plays an important role in providing neuroprotection, especially in ALS and Alzheimer’s disease. Nevertheless, the mechanisms underlying Sigma-1R activation and its effect on (G_4_C_2_)n-RNA-induced cell death remain unclear. In this study, we demonstrated that fluvoxamine is a Sigma-1R agonist that can increase chaperone activity and stabilize the protein expression of Pom121 in (G_4_C_2_)_31_-RNA-expressing NSC34 cells, leading to increased colocalization at the nuclear envelope. Interestingly, fluvoxamine treatment increased Pom121 protein expression without affecting transcription. In *C9orf72*-ALS, the nuclear translocation of TFEB autophagy factor decreased owing to nucleocytoplasmic transport defects. Our results showed that pretreatment of NSC34 cells with fluvoxamine promoted the shuttling of TFEB into the nucleus and elevated the expression of LC3-II compared to the overexpression of (G_4_C_2_)_31_-RNA alone. Additionally, even when used alone, fluvoxamine increases Pom121 expression and TFEB translocation. To summarize, fluvoxamine may act as a promising repurposed medicine for patients with *C9orf72*-ALS, as it stabilizes the nucleoporin Pom121 and promotes the translocation of TFEB in (G_4_C_2_)_31_-RNA-expressing NSC34 cells.

**Supplementary Information:**

The online version contains supplementary material available at 10.1007/s12035-023-03885-9.

## Introduction

*C9orf72* amyotrophic lateral sclerosis (ALS) is a familial ALS first identified in 2011 [1–3]. *C9orf72*-ALS is caused by a GGGGCC (G4C2) hexanucleotide repeat expansion (HRE) and the accumulation of five dipeptide repeat proteins (poly-PR, GR, PA, GP, and GA), resulting in motor or cognitive dysfunction [4, 5]. The deficits in the nucleocytoplasmic transport led to *C9orf72*-ALS progression through inhibiting Ras-related GTPase (RAN) and transcription factor (for example, TFEB) translocation [3, 6, 7]. Therefore, there is an urgent need to develop therapeutic agents that promote nucleocytoplasmic transport.

Autophagy dysregulation is recently indicated to cause the progression of ALS, especially *C9orf72*-ALS [7–9]. One mechanism involves the accumulation of the transcriptional regulator of autophagy/lysosomal function, TFEB, in the cytosol, owing to impaired nucleocytoplasmic transport function [7, 9]. Nucleoporin POM121 reportedly acts as a nuclear pore regulator that facilitates importin β1 and translocation of TFEB into the nucleus [7]. Because of the importance of POM121 in autophagy, further investigation into therapeutic approaches targeting POM121 in G4C2 RNA-induced NSC34 cellular models is required.

Sigma-1 receptor (Sig-1R) is a ligand-induced chaperone protein that has been demonstrated to exist in various cellular locations, including the mitochondria-endoplasmic reticulum (ER), nuclear envelope, and nuclear pore complex [6, 7, 10, 11]. Previous studies have demonstrated that Sig-1R agonists exert neuroprotective effects in various neurodegenerative diseases, such as ALS, Huntington’s disease, Parkinson’s disease, and Alzheimer’s disease [7, 12–14]. Fluvoxamine, a selective serotonin reuptake inhibitor, is also a high-affinity ligand of Sig-1R (Ki = 17.0 nM) [15, 16]. Fluvoxamine has been shown to exhibit a neuroprotective effect that prevents neuronal cell death resulting from ER stress [15] and decreases β-amyloid production by inhibiting γ-secretase activity [17]. However, the effects of fluvoxamine on autophagy through nucleocytoplasmic transport in G_4_C_2_ RNA-induced ALS require further investigation.

The present study showed that fluvoxamine functions as a Sig-1R agonist, as evidenced by its ability to decrease the association between BiP and Sig-1R and increase chaperone activity in a citrate synthase (CS) aggregation assay. Furthermore, when treated with fluvoxamine, there was an increase in the stabilization of nucleoporin Pom121 expression, both with and without (G_4_C_2_)_31_-RNA repeat stimulation. Subsequently, we observed that fluvoxamine treatment promoted the translocation of the autophagy transcription factor, TFEB, into the nucleus, resulting in increased LC3-II expression. These findings suggest that fluvoxamine can activate Sig-1R, elevate nucleoporin Pom121 expression, and enhance autophagy function in *C9orf72*-ALS.

## Materials and Methods

### Cell Culture and Transfection

The motor neuron-like cell line NSC34 was purchased from CELLutions Biosystems Inc. (CLU140). The cells were maintained in Dulbecco’s modified Eagle’s medium (DMEM; GIBCO, 11,965–092) supplemented with 10% fetal bovine serum (FBS) and 1% penicillin-streptomycin (GIBCO, 15,140–122). When the cell density reached 90% of a 10-cm dish, the cells were sub-cultured and seeded for gene transfection. Dishes (10 cm) and Poly-Jet reagent (SignaGen Laboratories, SL100688) were used, and the ratio of reagents to plasmids was 2:1. The mixture was incubated in serum-free DMEM (0.5 mL) for 20 min at 23 °C before being added to a 10-cm culture dish and incubated with the cells for the designated time.

### Cell Counting Kit-8 (CCK-8) Assay

Briefly [18], cells were treated with or without fluvoxamine at various time intervals. Following this, the culture media were removed from the experimental cells and replaced with a diluted Cell Counting Kit-8 (CCK-8) reagent (Abcam, ab228554) for 1 h. Any resulting crystallization was subsequently dissolved in the media. The solution was then measured spectrophotometrically at 450 nm using an ELISA plate reader.

### Cell Lysis and Protein Extraction

Cell pellets were incubated with ice-cold IP buffer (50 mM NaCl, 0.5% Nonidet P-40, 10 mM Tris-HCl, pH 8.0, and 1× protease inhibitor) for 30-min lysis. The lysate was centrifuged at 13,000 rpm for 10 min to extract proteins. The concentration of the extracted protein was measured using a Micro BCA™ Protein Assay Kit (Thermo Fisher Scientific Inc.).

### Immunoprecipitation (IP) Assay

The extracted protein was supplemented with the required amount of IP buffer and incubated with the IgG antibody or the designated primary antibody at 4 °C overnight. The mixture was then washed with IP buffer and incubated with sample buffer at 95 °C for 10 min. Finally, western blot analysis was performed.

### Nuclear-Cytoplasmic Fraction

A subcellular Protein Fractionation Kit for Cultured Cells (Thermo Fisher Scientific, 78840) was used to extract subcellular protein fractions from the harvested cells. All the reagents were mixed with protease inhibitors. Cytoplasmic extraction buffer was initially added to cell pellets, and the mixture was incubated at 4 °C. The resulting mixture was centrifuged to obtain the supernatant (cytosolic extraction). Next, membrane extraction buffer was added to the pellets before vortexing and being incubated at 4 °C. After obtaining the supernatant from the centrifugation of the complex, nuclear extraction buffer was added to the pellets, followed by centrifugation to obtain nuclear extraction.

### Cycloheximide (CHX) Chasing Assay

The old medium was discarded from the dish containing the cells, and fresh medium and CHX (100 μg/mL) were added. The cells were then incubated at 37 °C for an appropriate amount of time. Subsequently, the cells were collected and lysed to extract proteins for western blot analysis [7].

### Western Blot Analysis

Equal amounts of extracted protein were mixed with sample buffer before being denatured at 95 °C for 10 min. The proteins were then separated using sodium dodecyl-sulfate-polyacrylamide gel electrophoresis (SDS-PAGE) and electrotransferred onto a polyvinylidene difluoride (PVDF) membrane. The membrane was blocked with 5% skim milk in tris-buffered saline containing 0.1% Tween-20 (v/v; TBST) for 1 h. Subsequently, the blocked membranes were incubated with the solution of various primary antibodies diluted with TBST and incubated overnight at 4 °C. The membranes were washed thrice with TBST for 10 min and incubated with a solution of secondary antibodies diluted with TBST for 1 h. After washing thrice with TBST, the results were captured using an Azure 400 system.

### Chaperone Activity Assay

CS (1.1 mM; Sigma-Aldrich, C-3260) was incubated in 100 μL of 50 mM HEPES-NaOH buffer (pH 7.5) with GST or GST-SIGMAR1/Sigma-1 receptor (1 mM) at 45 °C in the presence or absence of fluvoxamine. Next, we monitored the scattered light at 320 nm for 70–90 min using a SpectraMax M2 (Molecular Devices, San Jose, CA, USA) detector [7].

### Immunostaining Assay

Cells were plated on poly L-lysine-coated coverslips and fixed with 4% paraformaldehyde. After permeabilization with 0.1% Triton X-100 in PBS, cells were washed with phosphate-buffered saline (PBS) and blocked with 10% serum diluted in PBS for 1 h. The cells were incubated with various primary antibodies overnight to detect the target proteins. The coverslips were washed with PBS and TBST before incubation with secondary antibodies in PBS for 1 h. After further washing with TBST and PBS, the cells were stained with 4′,6-diamidino-2-phenylindole (DAPI) solution to dye the chromatin, and images were captured using confocal microscopy.

### RNA Extraction and Reverse Transcription-Quantitative Polymerase Chain Reaction (RT-qPCR) Assay

RNA was extracted using an RNA extraction kit (RNeasy Mini Kit; Qiagen, Hilden, Germany). Experimental cells were lysed with lysis buffer, and the complex was supplemented with 70% ethanol. After purification, the RNA was collected and reverse-transcribed into DNA using an RT-qPCR kit [19]. The resulting cDNA was mixed with designed primers and enzymes in CFX96 Touch Real-Time PCR Detection System (BioRad). We determined the CT values and calculated the relative expression of the target genes [20].

### Statistical Analysis

**Fig. 1:**
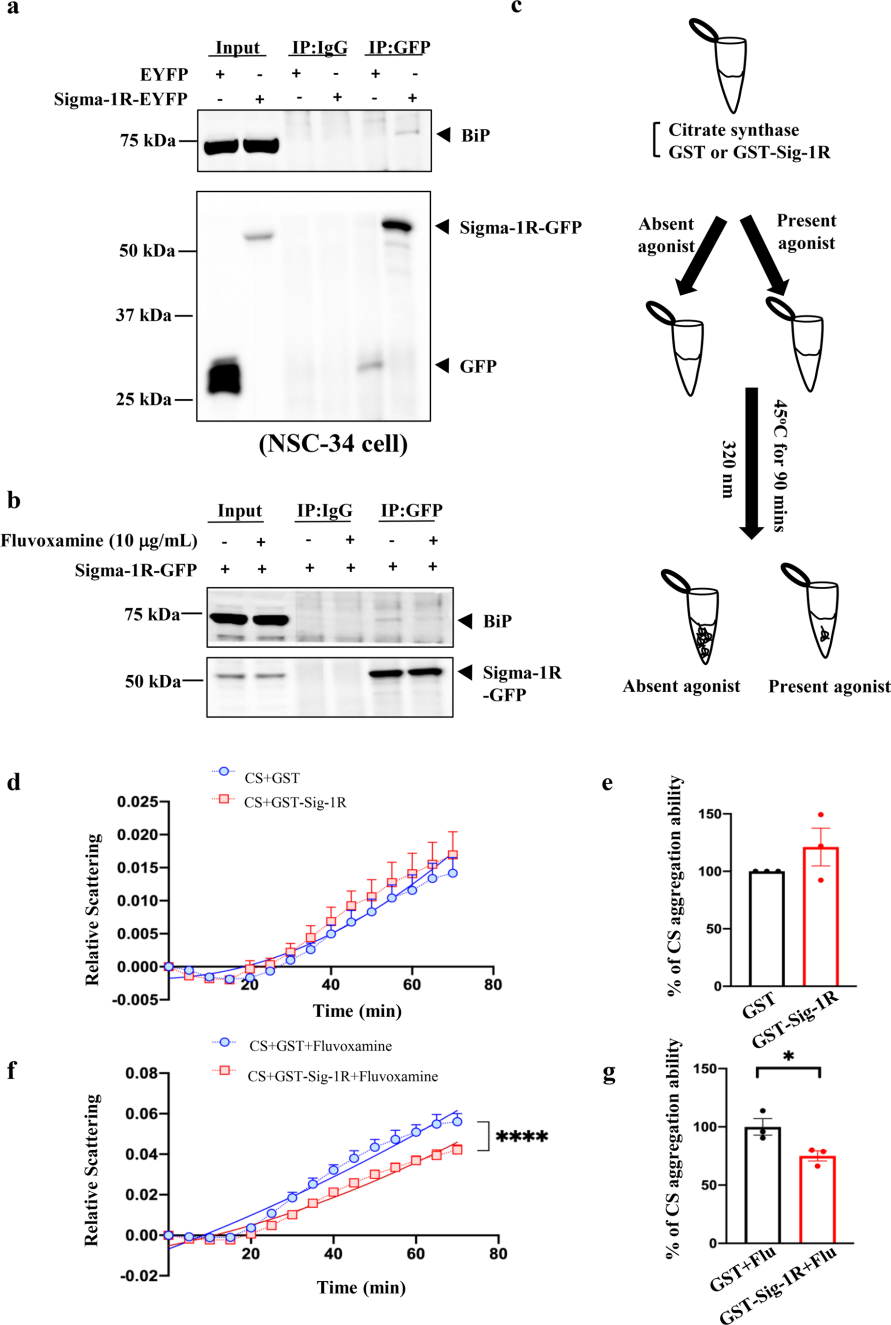
Sigma-1R was activated by fluvoxamine in NSC34 cells. **a** BiP interacted with Sigma-1R in NSC34 cells. We first transfected NSC34 cells with 5 μg EYFP or Sigma-1R-EYFP for 24 h. Cells were lysed to conduct IP to pull down GFP. Next, we performed western blotting using anti-BiP and anti-GFP as the primary antibodies. **b** Fluvoxamine treatment led to the dissociation of BiP from Sigma-1R in NSC34 cells. Cells were transfected with 5 μg Sigma-1R-EYFP and then treated with or without 10 μg/mL fluvoxamine for 1 h. Cells were lysed to conduct IP to pull down GFP. Subsequently, western blotting was performed, and anti-BiP and anti-GFP were used as the primary antibodies. **c** The illustration of chaperone activity assay of Sigma-1R supplemented with agonists. CS was mixed with either GST or GST-Sigma-1R in the presence of agonists or without in tubes. Then, the tubes were heated at 45 °C for 90 min. Finally, light scattering at 320 nm was measured. **d** The chaperone activity of Sigma-1R in the presence of CS. After the experiments as described in **c**, the results were analyzed using Prism (version 9.4.0). The relative scattering was expressed in arbitrary units, and the time length ranged from 0 to 70 min. The data are presented as means ± SEM, and a non-linear regression with the best fit was performed (*N* = 3). **e** Citrate aggregation ability was not significantly regulated by Sigma-1R after 70 min. Results of **d** were analyzed using the Student’s *t*-test and were considered statistically significant (*N* = 3). **f** Fluvoxamine significantly promoted the chaperone activity of Sigma-1R in the presence of CS. Following the experiments as described in **c**, results were analyzed using Prism software (version 9.4.0). Relative scattering was expressed in arbitrary units, and time length ranged from 0 to 70 min. The data are presented as means ± SEM, and a non-linear regression with the best fit was performed (*N* = 3). ****P < 0.0001 was considered statistically significant. **g** Fluvoxamine significantly enhanced the chaperone activity of Sigma-1R in NSC34 at 70 min. Results of **f** were analyzed using the Student’s *t*-test. *P < 0.05 was considered statistically significant (*N* = 3)

The data were collected from at least three independent experiments and analyzed using Prism software (version 9.4.0). The results are presented as the means ± standard error of the mean (SEM). Statistical significance was determined using unpaired Student’s *t*-test, one-way analysis of variance (ANOVA), or two-way ANOVA, along with a proper post hoc test. For comparisons between nonlinear regression curves (Fig. 1d–f), the second-order polynomial (quadratic) model was first used for curve fitting. Next, the “extra sum-of-squares *F*-test” was used to test if the best-fit curve of a group is the same as the global (shared) fitting curve. Statistical significance was set at *P* < 0.05.

## Results

### Fluvoxamine Acted as an Agonist to Enhance the Chaperone Ability of Sig-1R

First, NSC34 cells were transfected with Sig-1R to evaluate the association between BiP and Sig-1R. These results indicated that the interaction between Sig-1R and BiP was enhanced by the overexpression of Sig-1R-EYFP, consistent with previous reports implying that Sig-1R dissociates from BiP when activated (Fig. 1a) [21]. We performed IP to determine the effect of fluvoxamine on the BiP/Sig-1R interaction and found a significant decrease in the association of Sig-1R with BiP in NSC34 cells transfected with Sig-1R-EYFP and treated with 10 μg/mL fluvoxamine for 1 h (Fig. 1b). CS, a thermosensitive enzyme, is commonly used to assess chaperone ability [22]. We investigated the chaperone function by the analysis of CS together with GST-Sig-1R and fluvoxamine at 45 °C for 90 min (Fig. 1c). Data represented that GST-Sig-1R alone did not prevent CS aggregation at 70 min (Fig. 1d, e). However, treatment with 10 μg/mL fluvoxamine and Sig-1R significantly reduced CS aggregation by facilitating the chaperone ability of Sig-1R, compared to treatment with 10 μg/mL fluvoxamine alone (Fig. 1f, g). We further examined the cytotoxicity of fluvoxamine treatment in NSC34 motor neuron-like cells. The results showed that fluvoxamine did not affect cell viability at various time points after treatment (Supplementary fig. 1).

### Nucleoporin Pom121 Protein Expression Was Increased by Fluvoxamine in NSC34 Cells

**Fig. 2:**
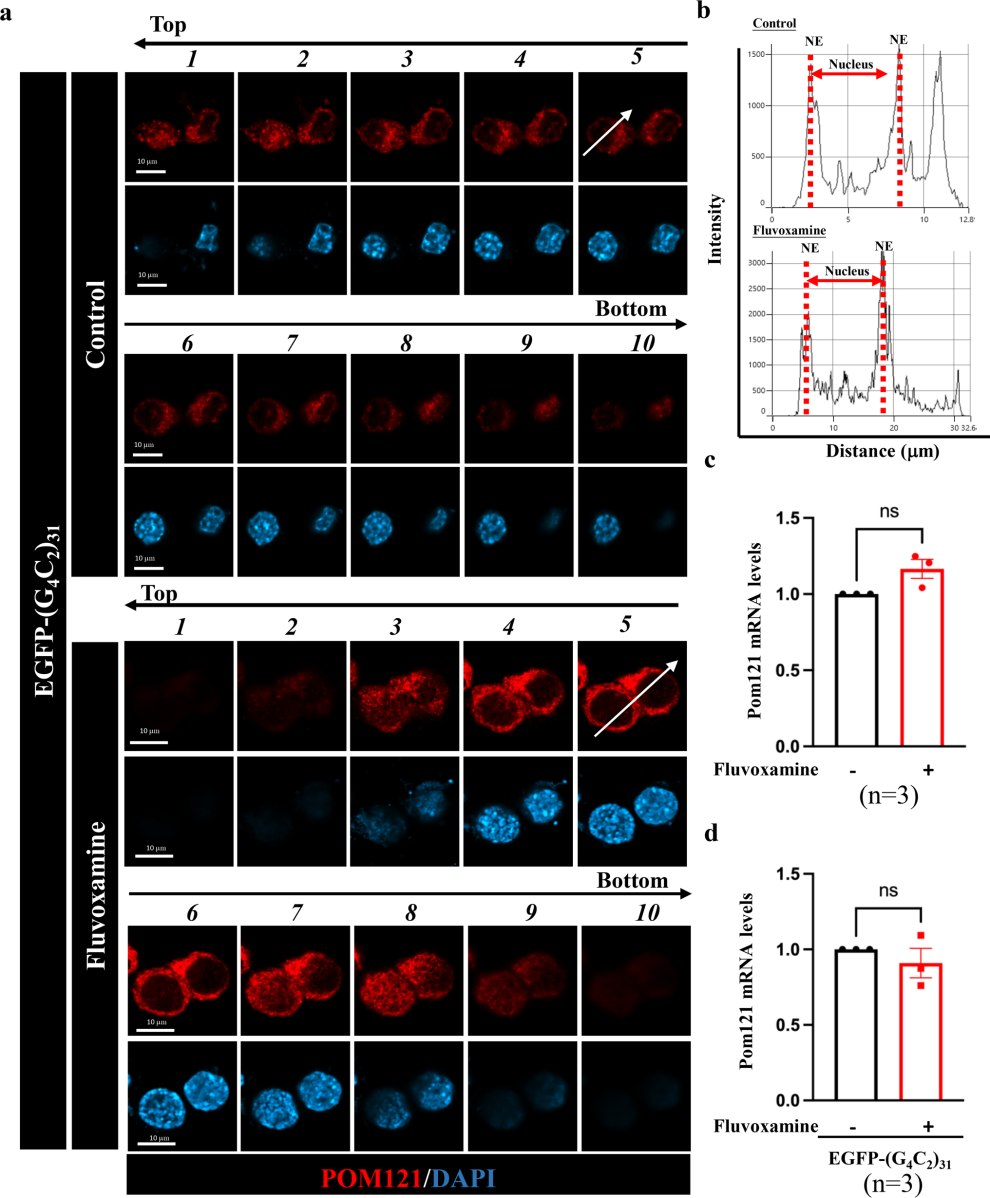
Fluvoxamine upregulated the expression of Pom121 under the stimulation of G4C2 repeat RNA not through transcriptional level. **a** We treated NSC34 cells transfected with EGFP-(G_4_C_2_)_31_ with fluvoxamine, as represented by the z-stacks. We initially added 10 μg/mL fluvoxamine to NSC34 cells for 1 h, followed by transfection with EGFP-(G_4_C_2_)_31_ for 24 h. Next, immunostaining was performed using anti-Pom121 and DAPI. The results were photographed using confocal microscopy and are presented as z-stacks from the top to the bottom of the cells. **b** Fluvoxamine increased the localization of Pom121 in the nuclear envelope of NSC34 cells in the 5th z-stack. This was determined by analyzing the data from **a** using ImageJ software along the arrows of the 5th z-stack and comparing it to the control group’s data. **c** Fluvoxamine treatment alone did not affect Pom121 transcription levels in NSC34 cells. RNA extraction and RT-qPCR were conducted to measure the expression of Pom121. After calculating in 2^−ΔΔCT^, data were analyzed using Prism software and the Student’s *t*-test. **d** Fluvoxamine did not significantly affect the mRNA expression level of Pom121 in NSC34 cells. Cells were first supplemented with or without 10 μg/mL fluvoxamine for 1 h. Then, the cells were transfected with EGFP-(G_4_C_2_)_31_ for 24 h. Thereafter, RNA extraction and RT-qPCR were conducted to measure the expression of Pom121. After calculating in 2^−ΔΔCT^, data were analyzed using Prism software and the Student’s *t*-test. *P < 0.05 was considered statistically significant (*N* = 3)

Previous studies have shown that Pom121 is not regulated by EGFP-(G_4_C_2_)_31_ and that Sigma-1R can chaperone Pom121, which participates in the nucleocytoplasmic transport of transcription factors [7, 23]. In Fig. 1, we confirmed fluvoxamine as a Sigma-1R agonist. Therefore, to investigate whether fluvoxamine plays a role in *C9orf72-*ALS and affects the nuclear pore complex (NPC), we pretreated NSC34 cells with 10 μg/mL fluvoxamine and overexpressed (G_4_C_2_)_31_ RNA in NSC34 cells, followed by immunostaining and RT-qPCR. Z-stacks of images of fluorescently stained NSC34 cells transfected with EGFP-(G_4_C_2_)_31_ are shown in the aerial view in Fig. 2a. Tracking of the signal intensities in the 5th stack suggested that Pom121 (red) was mainly located at the nuclear envelope in NSC34 cells treated with fluvoxamine compared to the control group (arrows shown in Fig. 2b). The Pom121 mRNA transcription levels did not show a significant difference after fluvoxamine treatment alone (Fig. 2c). Additionally, the Pom121 mRNA levels had no difference between NSC34 cells treated with EGFP-(G_4_C_2_)_31_ plus fluvoxamine and EGFP-(G_4_C_2_)_31_ alone (Fig. 2d, *N* = 3).

### Pom121 Was Stabilized Through Supplementation with Fluvoxamine in NSC34 Cells

**Fig. 3:**
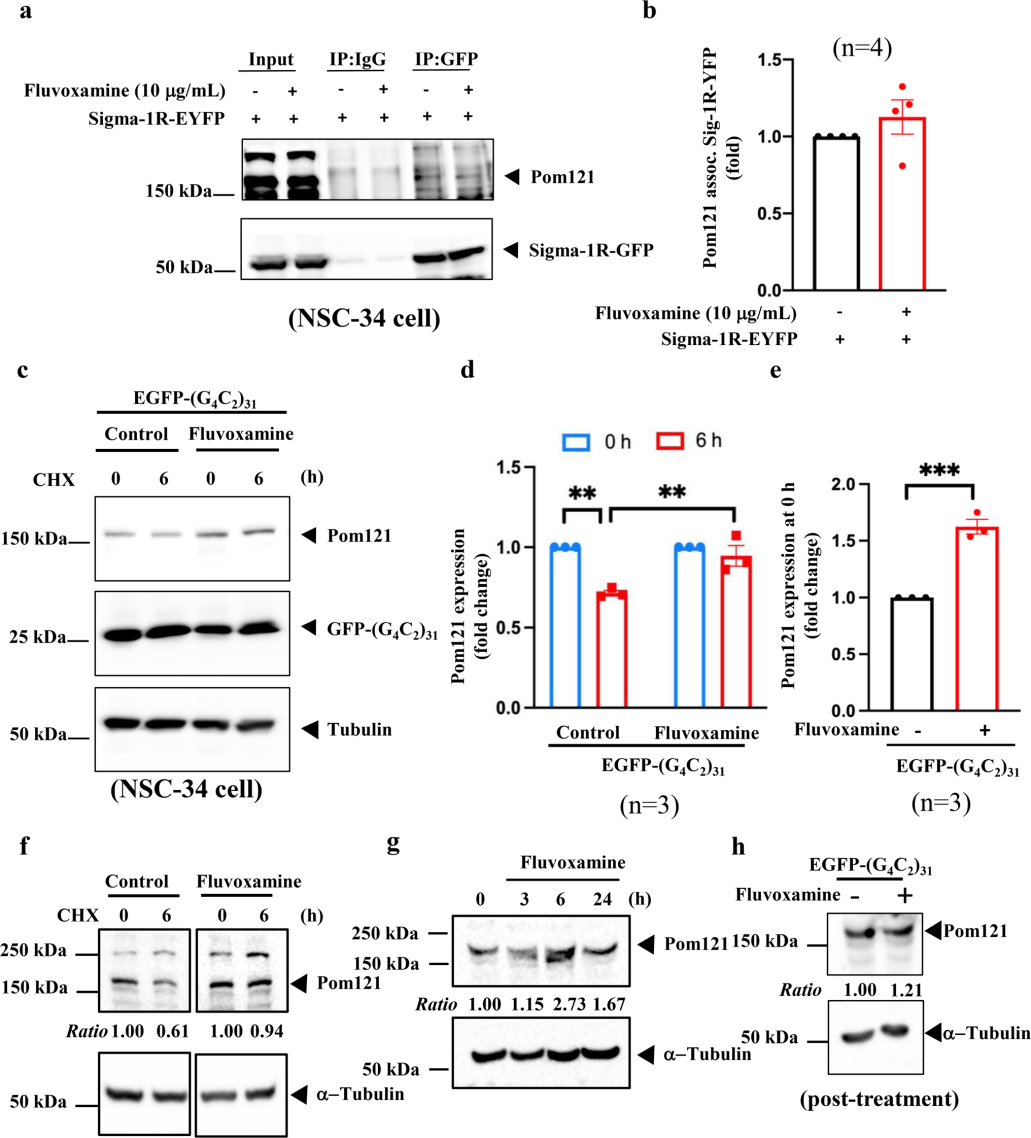
Fluvoxamine stabilized the expression of Pom121 without interfering with the association with Sigma-1R. **a** NSC34 cells pretreated with or without 10 μg/mL fluvoxamine for 1 h were overexpressed with Sigma-1R-EYFP for 24 h. Protein extraction and western blotting were performed using anti-Pom121 and anti-GFP as the primary antibodies. **b** The statistical results of **a**. Results were analyzed using ImageJ software and Prism using the Student’s *t*-test. *P < 0.05 was considered statistically significant (*N* = 4). **c** NSC34 cells were initially treated with 10 μg/mL fluvoxamine for 1 h and transfected with EGFP-(G_4_C_2_)_31_ for 24 h. The cells were supplemented with 100 μg/mL CHX for 0 or 6 h, followed by protein extraction and western blot analysis using anti-Pom121 and anti-GFP as the primary antibodies. We finally quantified the results using ImageJ software. **d** The statistical results of **c**. The data at 0 and 6 h were analyzed using one-way ANOVA. **P < 0.01 was considered statistically significant (*N* = 3). **e** The statistical results of **c**. Data at 0 were analyzed using the two-tailed unpaired Student’s *t*-test with Prism software. ***P < 0.001 was considered statistically significant (*N* = 3). **f** Fluvoxamine treatment alone stabilizes Pom121 expression in NSC34 cells. NSC34 cells were treated with 10 μg/mL of fluvoxamine for 24 h. After the initial treatment, the cells were supplemented with 100 μg/mL of CHX for either 0 or 6 h. Subsequently, protein extraction was performed, and western blot analysis was conducted using anti-Pom121 and anti-GFP as primary antibodies. Finally, the results were quantified using ImageJ software. **g** Fluvoxamine treatment alone increased Pom121 protein expression. The expression levels of Pom121 were observed to increase upon treatment with fluvoxamine. **h** NSC34 cells were transfected with EGFP-(G_4_C_2_)_31_ for 24 h. Subsequently, the transfected cells will treat with fluvoxamine for 24 h. The protein extraction was performed, and western blot analysis was conducted using anti-Pom121

Based on the aforementioned results, we examined the connection between Sigma-1R, Pom121, and fluvoxamine. IP data revealed that Sigma-1R interacted with Pom121 in NSC34 cells overexpressing Sigma-1-EYFP, with or without treatment with 10 μg/mL fluvoxamine (Fig. 3a, b). As shown in Fig. 2d, at the transcriptional level, Pom121 expression was not altered by EGFP-(G4C2)_31_. We used 100 μg/mL CHX, a protein translation inhibitor, to assess Pom121 stability. Western blotting demonstrated that fluvoxamine stabilized Pom121 in NSC34 cells transfected with EGFP-(G_4_C_2_)_31_ (Fig. 3c, d, control group vs. fluvoxamine group at 6 h, **P < 0.01, *N* = 3). Pom121 protein expression was notably increased in the fluvoxamine group at 0 h (Fig. 3e, **P < 0.01, *N* = 3). As well, the protein stability levels of Pom121 were increased in the treatment of fluvoxamine alone (Fig. 3f). We performed the ubiquitination assay to assess whether fluvoxamine can decrease the ubiquitination of Pom121. Our results suggest that fluvoxamine did not have an impact on the ubiquitination of Pom121. Consequently, we hypothesize that the stabilized expression of Pom121 protein occurs via a non-ubiquitin proteasome pathway (Supplementary fig. 2). Further, we assessed the expression levels of Pom121, which were increased by treatment of fluvoxamine alone at 6 and 24 h (Fig. 3g). Finally, we also examined whether the expression levels of Pom121 could be regulated by post-treatment with fluvoxamine in EGFP-(G_4_C_2_)_31_-expressing NSC34 cells. The data showed a slight increase in Pom121 expression following post-treatment with fluvoxamine in EGFP-(G_4_C_2_)_31_-expressing NSC34 cells (Fig. 3h). Based on these findings, fluvoxamine enhanced Pom121 expression by stabilizing its protein turnover in NSC34 cells.

### Treatment of Fluvoxamine in NSC34 Cells Promoted Autophagy Through the Translocation of TFEB and Importin β1

**Fig. 4:**
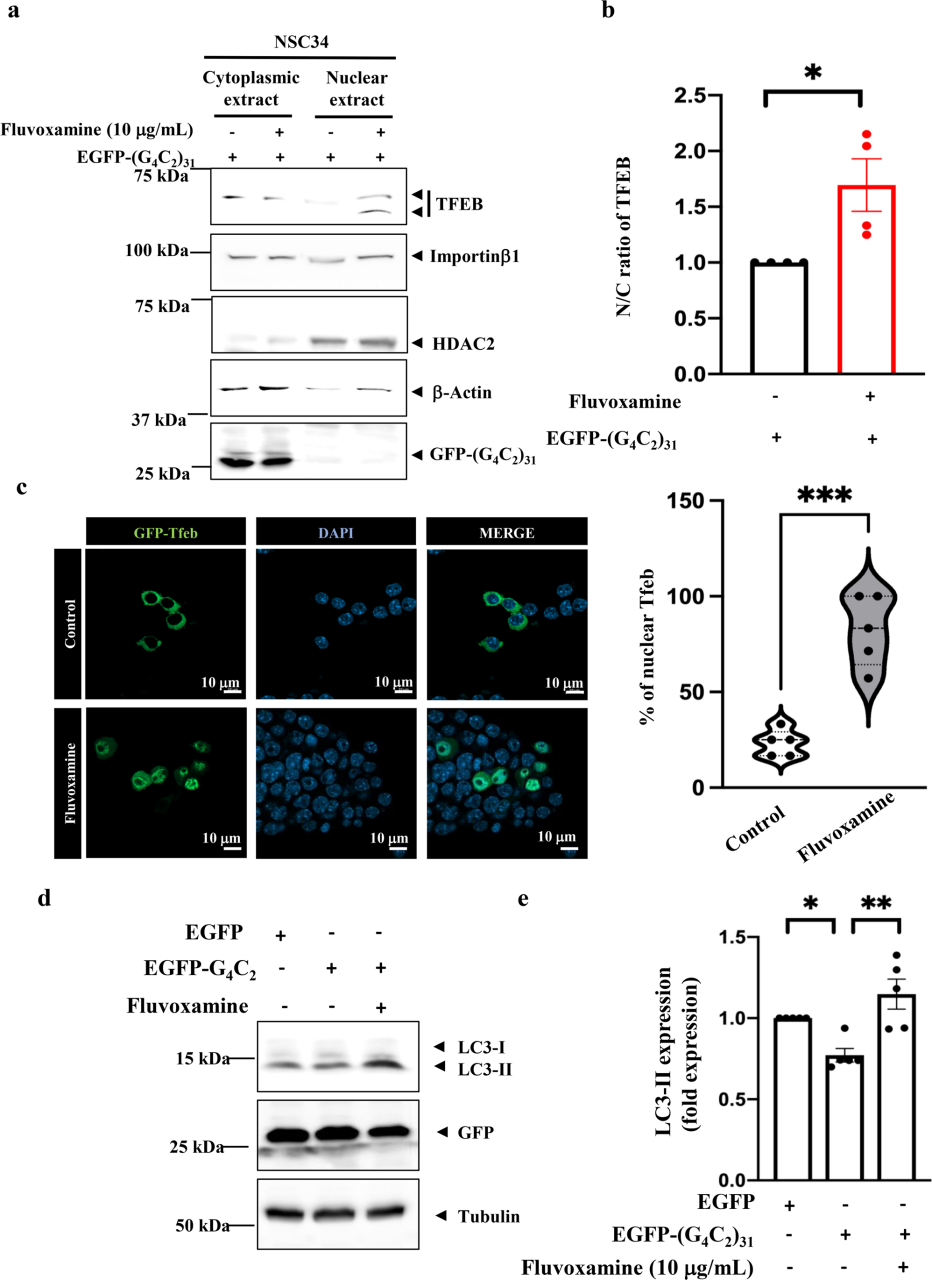
Fluvoxamine promoted autophagy by facilitating the nuclear import of TFEB together with importin β1 in NSC34 cells under the stimulation of G4C2 repeat RNA. **a** NSC34 cells pretreated with or without 10 μg/mL fluvoxamine for 1 h were subsequently overexpressed with EGFP-(G_4_C_2_)_31_ for 24 h. Nuclear-cytoplasmic fraction, as well as western blotting, was then performed. Anti-TFEB, anti-importin β1, anti-HDAC2 (as the internal control of nucleoplasm), anti-β-actin (as the internal control of cytosol), and anti-GFP were used as the primary antibodies. The results were quantified using ImageJ software. **b** The statistical results of **a**. Data were calculated as the ratios of two nuclear TFEB bands to cytosol TFEB, which were analyzed using the unpaired Student’s *t*-test. The two nuclear TFEB bands were normalized using HDAC2 as the nuclear internal control, while cytosolic TFEB was normalized with β-actin. *P < 0.05 was considered statistically significant (*N* = 4). **c** Fluvoxamine treatment alone promoted GFP-TFEB translocation into the nucleus. Confocal images revealed the GFP-TFEB expression (green) in NSC34 cells. The quantification of data from the left panel showed a significant increase in the intensity of nuclear GFP-TFEB. The percentage of nuclear TFEB was performed by using NIH ImageJ. **d** NSC34 cells pretreated with or without 10 μg/mL fluvoxamine for 1 h were overexpressed with EGFP-(G_4_C_2_)_31_ or GFP for 24 h. Protein extraction and western blotting were then performed. Anti-LC3, anti-GFP, and anti-tubulin were used as the primary antibodies. The results were quantified using ImageJ software. **e** The statistical results of **d**. Data were analyzed using one-way ANOVA. *P < 0.05 and **P < 0.01 were considered statistically significant (*N* = 5)

Autophagy defects are linked to importin β1 during nuclear import and are involved in the pathogenesis of *C9orf72*-ALS [7]. Previous studies have shown that importin β1, together with Pom121, participates in the nuclear translocation of TFEB, a transcription factor regulating the expression of autophagy and lysosomal biogenesis-related genes. G_4_C_2_ repeat RNA alters the nuclear-to-cytoplasmic (N/C) ratio of TFEB [7]. In the present study, we investigated the role of fluvoxamine in the nucleocytoplasmic transport of TFEB and expression of LC3-II, which marks the formation of autophagosomes in NSC34 cells overexpressing G_4_C_2_ repeat RNA [7]. The results of the subcellular fraction assays suggested that fluvoxamine reversed the N/C ratio of TFEB in (G_4_C_2_)-RNA-expressing NSC34 cells (Fig. 4a, b, *P < 0.05, *N* = 4) (note: TFEB is typically recognized as a two-band entity, with phosphorylated TFEB (high molecular weight) and dephosphorylated TFEB (low molecular weight) being detected in the nucleus [7, 24]). We also demonstrated that the nuclear GFP-TFEB was significantly increased after fluvoxamine treatment alone (Fig. 4c). In addition, the western blotting data demonstrated EGFP-(G_4_C_2_)_31_ decreased the expression of LC3-II, and fluvoxamine significantly restored the expression of LC3-II in EGFP-(G_4_C_2_)_31_-expressing NSC34 cells (Fig. 4d, e, **P < 0.01, *N* = 5). As well, the treatment of fluvoxamine alone can increase LC3-II expression (Supplementary fig. 3). However, the lysosomal membrane protein Lamp2a showed no difference after fluvoxamine treatment (Supplementary fig. 4). Collectively, these findings revealed the role of fluvoxamine in regulating autophagy in motor neurons. However, it is important to note that other underlying mechanisms of fluvoxamine involved in this process still need to be explored.

**Fig. 5:**
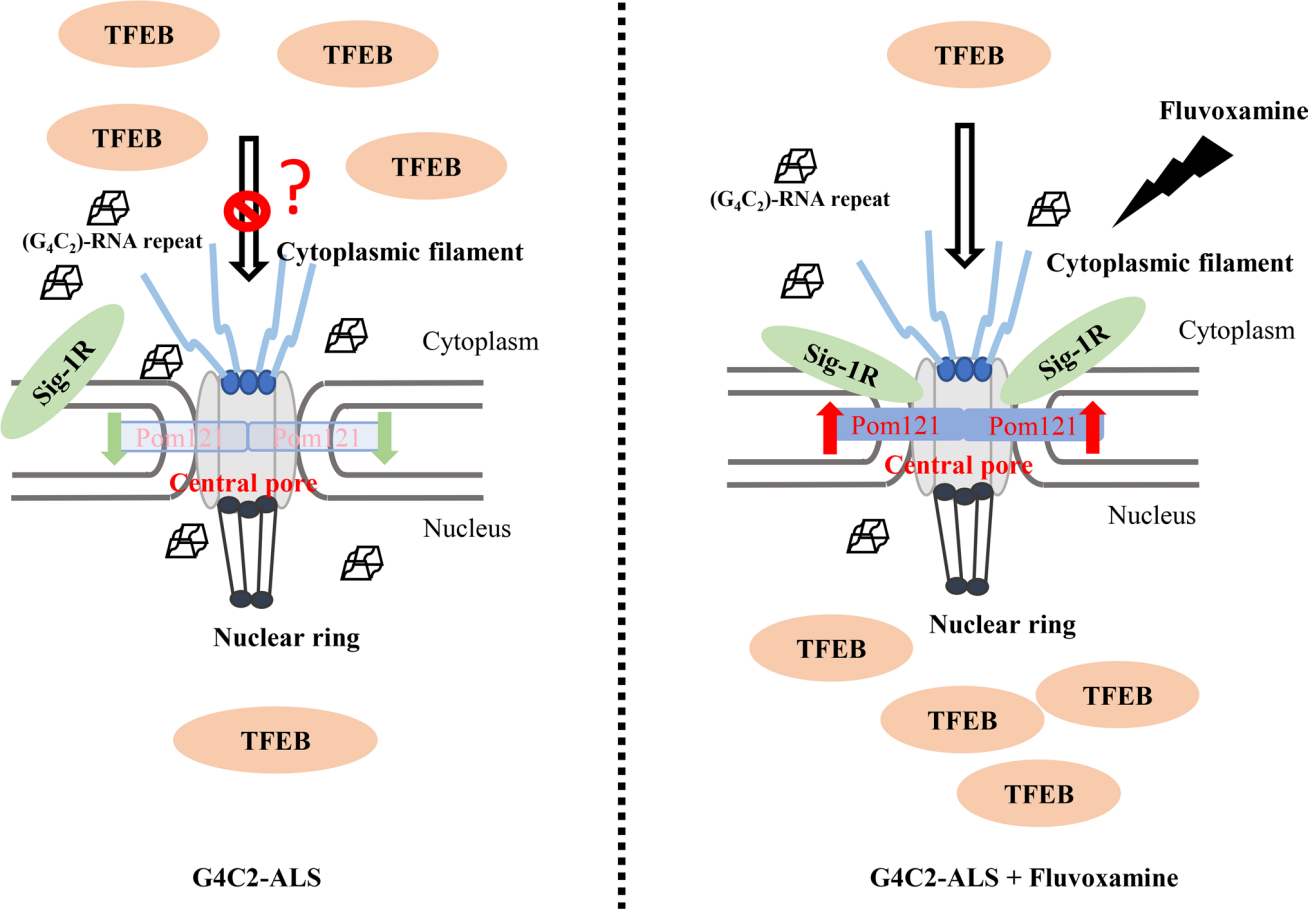
The schematic diagram of the proposed model. Fluvoxamine functioned as a Sigma-1R agonist to facilitate the import of TFEB into the nucleus through activation of Sigma-1R and upregulation of Pom121 expression. This reversed the defective autophagy observed in *C9orf72*-ALS induced by G4C2 repeat RNA

In summary, this study indicated that fluvoxamine acts as an agonist to dissociate Sigma-1R from BiP and enhances the chaperone activity of Sigma-1R. Moreover, fluvoxamine upregulated the expression of Pom121 by inhibiting protein degradation without influencing the interaction between Pom121 and Sigma-1R. Shuttling of TFEB and importin β1 into the nucleus was promoted by fluvoxamine in NSC34 cells. Similarly, LC3-II expression was upregulated through the stimulation of G_4_C_2_ repeat RNA along with fluvoxamine compared to the control group. Overall, the current study suggests that fluvoxamine promotes the translocation of TFEB into the nucleus and may serve as a therapeutic candidate for *C9orf72*-ALS (Fig. 5).

## Discussion

Several studies have indicated a noteworthy correlation between autophagy defects and the pathological progression of *C9orf72*-ALS in motoneurons [25–27]. Additionally, numerous experiments have demonstrated differentiating nuclear import under the stimulation of G_4_C_2_ repeat RNA, a toxic product from mutation of the *C9orf72* gene [7, 28]. According to the previous study, fluvoxamine dissociates Sigma-1R from BiP and boosts chaperone activity [15]. Furthermore, fluvoxamine increased the expression of Pom121, a crucial nucleoporin for nucleocytoplasmic shuttling, not by regulating its interaction with Sigma-1R but by enhancing the chaperone activity of Sigma-1R. Importantly, it influences the translocation of TFEB, a critical transcription factor for autophagy, from the cytoplasm to the nucleus, resulting in increased expression of LC3-II.

It has been reported that enhancing autophagy confers therapeutic advantages in Alzheimer’s disease (AD) and SARS-CoV-2 infection [29, 30]. Sigma-1R is activated by fluvoxamine and is associated with numerous neurodegenerative diseases, such as AD, Parkinson’s disease (PD), Huntington’s disease, and amyotrophic lateral sclerosis (ALS) [29]. Notably, fluvoxamine activates Sigma-1R, which in turn facilitates autophagy [29]. Autophagy dysregulation in motoneurons affected by *C9orf72* mutation led to neuronal death [7, 9, 26]. Our previous study showed that pridopidine, a Sigma-1R agonist, stabilizes nucleoporin Pom121 protein expression and subsequently increases the translocation of TFEB into the nucleus to promote autophagy in G_4_C_2_ repeat RNA-expressing NSC34 cells, resulting in reduced neuronal death [7]. Similarly, fluvoxamine, also a Sigma-1R agonist, stabilized Pom121 expression and promoted autophagy (Figs. 3 and 4). Repurposing existing drugs is important for treating various diseases, including neurodegenerative diseases. Fluvoxamine is a selective serotonin reuptake inhibitor widely used to treat depression [15]. Fluvoxamine treatment increased the transcription and protein expression of Sigma-1R in N2a cells [15]. These results suggest that fluvoxamine may increase Pom121 expression by increasing Sigma-1R levels (Fig. 3e), promoting nucleocytoplasmic transport. Fluvoxamine elevated Pom121 expression in Fig. 3e; nevertheless, the mRNA expression level of Pom121 was not regulated under the treatment of fluvoxamine (Fig. 2c and d). Sigma-1R has been demonstrated to affect 4E-BP1, which alters translational regulation after neuropathic pain [31]. Besides, it is important to recognize that the impact of fluvoxamine might extend beyond just the Sigma-1R, potentially encompassing other underlying molecules and pathways. Fluvoxamine has demonstrated strong inhibition of serotonin reuptake through the sodium-dependent serotonin transporter. Further exploration is required to investigate the connection between nucleoporin stabilization and sodium-dependent serotonin transporter [32, 33].

Notably, the nuclear pore complex is composed of approximately 30 nucleoporins, with Pom121 playing a critical role in regulating nucleocytoplasmic transport [7, 34]. Moreover, Pom121 can control the activity of other nucleoporins such as GP210, NDC1, Nup133, Nup107, Nup50, TPR, and Nup98 [34]. In the *C9orf72*-iPSN model, decreased Pom121 expression was observed, leading to disruption of nucleocytoplasmic transport [34]. Therefore, our study aimed to elucidate strategies for stabilizing or increasing Pom121 expression. Based on our previous study, Sigma-1R agonists may serve as potential therapeutic options for the treatment of *C9orf72*-ALS [7].

Because of the complex mechanism, TFEB translocates into the nucleus through the nuclear import cycle in which its nuclear localization signal (NLS) domain is conjugated with the importin α/β complex in the cytoplasm [7]. This complex then interacts with nucleoporins containing hydrophobic phenylalanine-glycine domain (FG domain), allowing TFEB to shuttle into nucleoplasm [7, 35, 36]. A previous study revealed that the reduction in Pom121 correlated with decreased intensity of FG-domain-containing Nups (FG-Nups) [37]. In the present study, we showed that Pom121 was stabilized by fluvoxamine and that nucleocytoplasmic transport was facilitated, as shown in Figs. 3c and 4a. This may indicate that Pom121 upregulation induced by Sigma-1R repairs the import of the cargo complex into the nucleus by restoring the number of FG-Nups in the central channel of the NPC. In future studies, we will further analyze FG-Nups in NSC34 cells transfected with Pom121 to elucidate the detailed mechanism. Once inside the nucleus, Ran-GTP interacts with and releases importin from TFEB, subsequently interacts with FG-Nups, and moves into the cytoplasm, where hydrolysis of Ran-GTP with the assistance of GTPase-accelerating protein (GAP) takes place [38]. Ran-GDP then translocates to the nucleoplasm [38]. In addition, Sigma-1R binds to FG-Nups and maintains their stability. It was also exhibited that Sigma-1R stabilizes RanGAP1, which is crucial for the recycling of Ran protein and the continuation of nucleocytoplasmic transport [6].

According to the previous study, TRAF2 and IRE1 are involved in the ER stress and the degeneration of fly eyes resulting from overexpression of (G4C2)-RNA [39]. Moreover, inhibition of IRE1 or TRAF2 would mitigate C9orf72 toxicity [39]. Separate studies also demonstrated that fluvoxamine displays neuroprotective effects through alleviating ER stress-mediated apoptosis [15, 40]. Thus, we speculate that fluvoxamine treatment could potentially protect motoneurons from (G4C2)-RNA-induced ER stress.

Regarding other toxic products in *C9orf72*-ALS, dipeptide repeats (DPRs) are caused by mutations in the *C9orf72* gene and contribute to interference in normal neuronal metabolism, such as the death of neurons and defects in nucleocytoplasmic shuttling [41, 42]. It has been suggested that autophagy is responsible for the clearance of DPR; however, G_4_C_2_ repeat RNA changes the process by retaining TFEB outside the nuclei [26]. This implied an irreversible accumulation of DPRs. Collectively, we hypothesize that fluvoxamine may activate Sigma-1R, chaperoning the Pom121 protein to reverse the impaired nuclear import and export. This consequently induces autophagy activation and potentially protects motor neurons from DPR toxicity.

In conclusion, fluvoxamine exerts its innate function to activate Sigma-1R and rescues the impaired distribution of TFEB in the cytoplasm and nucleus by modulating the expression of Pom121. These factors, in turn, contribute to repaired autophagy in (G_4_C_2_) RNA repeat-induced *C9orf72*-ALS. The neuroprotective ability of fluvoxamine may mediate the progression of numerous diseases, such as ALS, Parkinson’s disease, and Huntington’s disease. In this study, we validated the critical relationship between Sigma-1R and Pom121, as well as their ability to affect nucleocytoplasmic transport (Fig. 5). Moreover, we determined that fluvoxamine may act as a potential repurposed drug to prevent motoneurons from insulting *C9orf72*-ALS through its selective role as a Sigma-1R agonist.

### Supplementary Information

ESM 1 (DOCX 1797 kb)

## Data Availability

The data that support this study are available from the corresponding author upon reasonable request.

## Abbreviations

ALS: Amyotrophic lateral sclerosis
FTD: Frontotemporal dementia
G4C2: GGGGCC
NSC34: Mouse motor neuron-like hybrid cell line
HRE: Hexanucleotide repeat expansion
RAN: Ras-related GTPase
TFEB: Transcription factor EB
Sig-1R: Sigma-1 receptor
CS: Citrate synthase
NPC: Nuclear pore complex
CHX: Cycloheximide
PVDF: Polyvinylidene difluoride
SDS-PAGE: SDS-polyacrylamide gel electrophoresis
FG domain: Phenylalanine-glycine domain
DPRs: Dipeptide repeats
CCK-8: Cell Counting Kit-8

## References

[1] Gijselinck I, Van Langenhove T, van der Zee J, Sleegers K, Philtjens S, Kleinberger G, Janssens J, Bettens K, Van Cauwenberghe C, Pereson S, Engelborghs S, Sieben A, De Jonghe P, Vandenberghe R, Santens P, De Bleecker J, Maes G, Baumer V, Dillen L, Joris G, Cuijt I, Corsmit E, Elinck E, Van Dongen J, Vermeulen S, Van den Broeck M, Vaerenberg C, Mattheijssens M, Peeters K, Robberecht W, Cras P, Martin JJ, De Deyn PP, Cruts M, Van Broeckhoven C (2012). A C9orf72 promoter repeat expansion in a Flanders-Belgian cohort with disorders of the frontotemporal lobar degeneration-amyotrophic lateral sclerosis spectrum: a gene identification study. Lancet Neurol.

[2] Renton AE, Majounie E, Waite A, Simon-Sanchez J, Rollinson S, Gibbs JR, Schymick JC, Laaksovirta H, van Swieten JC, Myllykangas L, Kalimo H, Paetau A, Abramzon Y, Remes AM, Kaganovich A, Scholz SW, Duckworth J, Ding J, Harmer DW, Hernandez DG, Johnson JO, Mok K, Ryten M, Trabzuni D, Guerreiro RJ, Orrell RW, Neal J, Murray A, Pearson J, Jansen IE, Sondervan D, Seelaar H, Blake D, Young K, Halliwell N, Callister JB, Toulson G, Richardson A, Gerhard A, Snowden J, Mann D, Neary D, Nalls MA, Peuralinna T, Jansson L, Isoviita VM, Kaivorinne AL, Holtta-Vuori M, Ikonen E, Sulkava R, Benatar M, Wuu J, Chio A, Restagno G, Borghero G, Sabatelli M, Consortium I, Heckerman D, Rogaeva E, Zinman L, Rothstein JD, Sendtner M, Drepper C, Eichler EE, Alkan C, Abdullaev Z, Pack SD, Dutra A, Pak E, Hardy J, Singleton A, Williams NM, Heutink P, Pickering-Brown S, Morris HR, Tienari PJ, Traynor BJ (2011). A hexanucleotide repeat expansion in C9ORF72 is the cause of chromosome 9p21-linked ALS-FTD. Neuron.

[3] Zhang K, Donnelly CJ, Haeusler AR, Grima JC, Machamer JB, Steinwald P, Daley EL, Miller SJ, Cunningham KM, Vidensky S, Gupta S, Thomas MA, Hong I, Chiu SL, Huganir RL, Ostrow LW, Matunis MJ, Wang J, Sattler R, Lloyd TE, Rothstein JD (2015). The C9orf72 repeat expansion disrupts nucleocytoplasmic transport. Nature.

[4] Czuppa M, Dhingra A, Zhou Q, Schludi C, Konig L, Scharf E, Farny D, Dalmia A, Tager J, Castillo-Lizardo M, Katona E, Mori K, Aumer T, Schelter F, Muller M, Carell T, Kalliokoski T, Messinger J, Rizzu P, Heutink P, Edbauer D (2022). Drug screen in iPSC-neurons identifies nucleoside analogs as inhibitors of (G(4)C(2))(n) expression in C9orf72 ALS/FTD. Cell Rep.

[5] Gleixner AM, Verdone BM, Otte CG, Anderson EN, Ramesh N, Shapiro OR, Gale JR, Mauna JC, Mann JR, Copley KE, Daley EL, Ortega JA, Cicardi ME, Kiskinis E, Kofler J, Pandey UB, Trotti D, Donnelly CJ (2022). NUP62 localizes to ALS/FTLD pathological assemblies and contributes to TDP-43 insolubility. Nat Commun.

[6] Lee PT, Lievens JC, Wang SM, Chuang JY, Khalil B, Wu HE, Chang WC, Maurice T, Su TP (2020). Sigma-1 receptor chaperones rescue nucleocytoplasmic transport deficit seen in cellular and Drosophila ALS/FTD models. Nat Commun.

[7] Wang SM, Wu HE, Yasui Y, Geva M, Hayden M, Maurice T, Cozzolino M, Su TP (2023). Nucleoporin POM121 signals TFEB-mediated autophagy via activation of SIGMAR1/sigma-1 receptor chaperone by pridopidine. Autophagy.

[8] Wang H, Wang R, Xu S, Lakshmana MK (2016). Transcription factor EB is selectively reduced in the nuclear fractions of Alzheimer’s and amyotrophic lateral sclerosis brains. Neurosci J.

[9] Cunningham KM, Maulding K, Ruan K, Senturk M, Grima JC, Sung H, Zuo Z, Song H, Gao J, Dubey S, Rothstein JD, Zhang K, Bellen HJ, Lloyd TE (2020) TFEB/Mitf links impaired nuclear import to autophagolysosomal dysfunction in C9-ALS. Elife 9. 10.7554/eLife.59419 10.7554/eLife.59419 PMC7758070 33300868

[10] Hayashi T, Su TP (2007). Sigma-1 receptor chaperones at the ER-mitochondrion interface regulate Ca(2+) signaling and cell survival. Cell.

[11] Mavlyutov TA, Yang H, Epstein ML, Ruoho AE, Yang J, Guo LW (2017). APEX2-enhanced electron microscopy distinguishes sigma-1 receptor localization in the nucleoplasmic reticulum. Oncotarget.

[12] Eddings CR, Arbez N, Akimov S, Geva M, Hayden MR, Ross CA (2019). Pridopidine protects neurons from mutant-huntingtin toxicity via the sigma-1 receptor. Neurobiol Dis.

[13] Estevez-Silva HM, Cuesto G, Romero N, Brito-Armas JM, Acevedo-Arozena A, Acebes A, Marcellino DJ (2022). Pridopidine promotes synaptogenesis and reduces spatial memory deficits in the Alzheimer’s disease APP/PS1 mouse model. Neurotherapeutics.

[14] Francardo V, Bez F, Wieloch T, Nissbrandt H, Ruscher K, Cenci MA (2014). Pharmacological stimulation of sigma-1 receptors has neurorestorative effects in experimental parkinsonism. Brain.

[15] Omi T, Tanimukai H, Kanayama D, Sakagami Y, Tagami S, Okochi M, Morihara T, Sato M, Yanagida K, Kitasyoji A, Hara H, Imaizumi K, Maurice T, Chevallier N, Marchal S, Takeda M, Kudo T (2014). Fluvoxamine alleviates ER stress via induction of sigma-1 receptor. Cell Death Dis.

[16] Hashimoto K (2015). Activation of sigma-1 receptor chaperone in the treatment of neuropsychiatric diseases and its clinical implication. J Pharmacol Sci.

[17] Kim WS, Fu Y, Dobson-Stone C, Hsiao JT, Shang K, Hallupp M, Schofield PR, Garner B, Karl T, Kwok JBJ (2018). Effect of fluvoxamine on amyloid-beta peptide generation and memory. J Alzheimers Dis.

[18] Huang CP, Liu LC, Lu HL, Shyr CR (2023). Effects of hepatocyte growth factor on porcine mammary cell growth and senescence. Biomedicine (Taipei).

[19] Wang CH, Wu HC, Hsu CW, Chang YW, Ko CY, Hsu TI, Chuang JY, Tseng TH, Wang SM (2022) Inhibition of MZF1/c-MYC axis by cantharidin impairs cell proliferation in glioblastoma. Int J Mol Sci 23(23). 10.3390/ijms232314727 10.3390/ijms232314727 PMC9740304 36499054

[20] Wang SM, Hsu JC, Ko CY, Wu HE, Hsiao YW, Wang JM (2023). Astrocytic Cebpd regulates pentraxin 3 expression to promote fibrotic scar formation after spinal cord injury. Mol Neurobiol.

[21] Hayashi T (2019). The sigma-1 receptor in cellular stress signaling. Front Neurosci.

[22] Ahrman E, Gustavsson N, Hultschig C, Boelens WC, Emanuelsson CS (2007). Small heat shock proteins prevent aggregation of citrate synthase and bind to the N-terminal region which is absent in thermostable forms of citrate synthase. Extremophiles.

[23] Lim KS, Wong RW (2018). Targeting nucleoporin POM121-importin beta axis in prostate cancer. Cell Chem Biol.

[24] Li C, Wang X, Li X, Qiu K, Jiao F, Liu Y, Kong Q, Liu Y, Wu Y (2019). Proteasome inhibition activates autophagy-lysosome pathway associated with TFEB dephosphorylation and nuclear translocation. Front Cell Dev Biol.

[25] Beckers J, Tharkeshwar AK, Van Damme P (2021). C9orf72 ALS-FTD: recent evidence for dysregulation of the autophagy-lysosome pathway at multiple levels. Autophagy.

[26] Boivin M, Pfister V, Gaucherot A, Ruffenach F, Negroni L, Sellier C, Charlet-Berguerand N (2020). Reduced autophagy upon C9ORF72 loss synergizes with dipeptide repeat protein toxicity in G4C2 repeat expansion disorders. EMBO J.

[27] Balendra R, Isaacs AM (2018). C9orf72-mediated ALS and FTD: multiple pathways to disease. Nat Rev Neurol.

[28] Frottin F, Perez-Berlanga M, Hartl FU, Hipp MS (2021) Multiple pathways of toxicity induced by C9orf72 dipeptide repeat aggregates and G(4)C(2) RNA in a cellular model. Elife 10. 10.7554/eLife.62718 10.7554/eLife.62718 PMC8221807 34161229

[29] Prasanth MI, Malar DS, Tencomnao T, Brimson JM (2021). The emerging role of the sigma-1 receptor in autophagy: hand-in-hand targets for the treatment of Alzheimer’s. Expert Opin Ther Targets.

[30] Brimson JM, Prasanth MI, Malar DS, Brimson S, Thitilertdecha P, Tencomnao T (2021). Drugs that offer the potential to reduce hospitalization and mortality from SARS-CoV-2 infection: the possible role of the sigma-1 receptor and autophagy. Expert Opin Ther Targets.

[31] Wang SM, Goguadze N, Kimura Y, Yasui Y, Pan B, Wang TY, Nakamura Y, Lin YT, Hogan QH, Wilson KL, Su TP, Wu HE (2021). Genomic action of sigma-1 receptor chaperone relates to neuropathic pain. Mol Neurobiol.

[32] Sukhatme VP, Reiersen AM, Vayttaden SJ, Sukhatme VV (2021). Fluvoxamine: a review of its mechanism of action and its role in COVID-19. Front Pharmacol.

[33] Brimson JM, Brimson S, Chomchoei C, Tencomnao T (2020). Using sigma-ligands as part of a multi-receptor approach to target diseases of the brain. Expert Opin Ther Targets.

[34] Coyne AN, Zaepfel BL, Hayes L, Fitchman B, Salzberg Y, Luo EC, Bowen K, Trost H, Aigner S, Rigo F, Yeo GW, Harel A, Svendsen CN, Sareen D, Rothstein JD (2020). G(4)C(2) repeat RNA initiates a POM121-mediated reduction in specific nucleoporins in C9orf72 ALS/FTD. Neuron.

[35] Cingolani G, Petosa C, Weis K, Muller CW (1999). Structure of importin-beta bound to the IBB domain of importin-alpha. Nature.

[36] Dickmanns A, Kehlenbach RH, Fahrenkrog B (2015). Nuclear pore complexes and nucleocytoplasmic transport: from structure to function to disease. Int Rev Cell Mol Biol.

[37] Antonin W, Franz C, Haselmann U, Antony C, Mattaj IW (2005). The integral membrane nucleoporin pom121 functionally links nuclear pore complex assembly and nuclear envelope formation. Mol Cell.

[38] Wente SR, Rout MP (2010). The nuclear pore complex and nuclear transport. Cold Spring Harb Perspect Biol.

[39] Sahana TG, Chase KJ, Liu F, Lloyd TE, Rossoll W, Zhang K (2023). c-Jun N-terminal kinase promotes stress granule assembly and neurodegeneration in C9orf72-mediated ALS and FTD. J Neurosci.

[40] Tanimukai H, Kudo T (2015). Fluvoxamine alleviates paclitaxel-induced neurotoxicity. Biochem Biophys Rep.

[41] Xu W, Xu J (2018). C9orf72 dipeptide repeats cause selective neurodegeneration and cell-autonomous excitotoxicity in drosophila glutamatergic neurons. J Neurosci.

[42] Ryan S, Rollinson S, Hobbs E, Pickering-Brown S (2022). C9orf72 dipeptides disrupt the nucleocytoplasmic transport machinery and cause TDP-43 mislocalisation to the cytoplasm. Sci Rep.

