# Supplementary material for: Fluvoxamine Exerts Sigma-1R to Rescue Autophagy via Pom121-Mediated Nucleocytoplasmic Transport of TFEB
Source: Mol Neurobiol. 2024 Jan 5;61(8):5282–94. doi: 10.1007/s12035-023-03885-9 (PMC11249700; doi:10.1007/s12035-023-03885-9)
Supplement: Supplementary file 1 — (DOCX 1797 kb) [file 12035_2023_3885_MOESM1_ESM.docx]

**
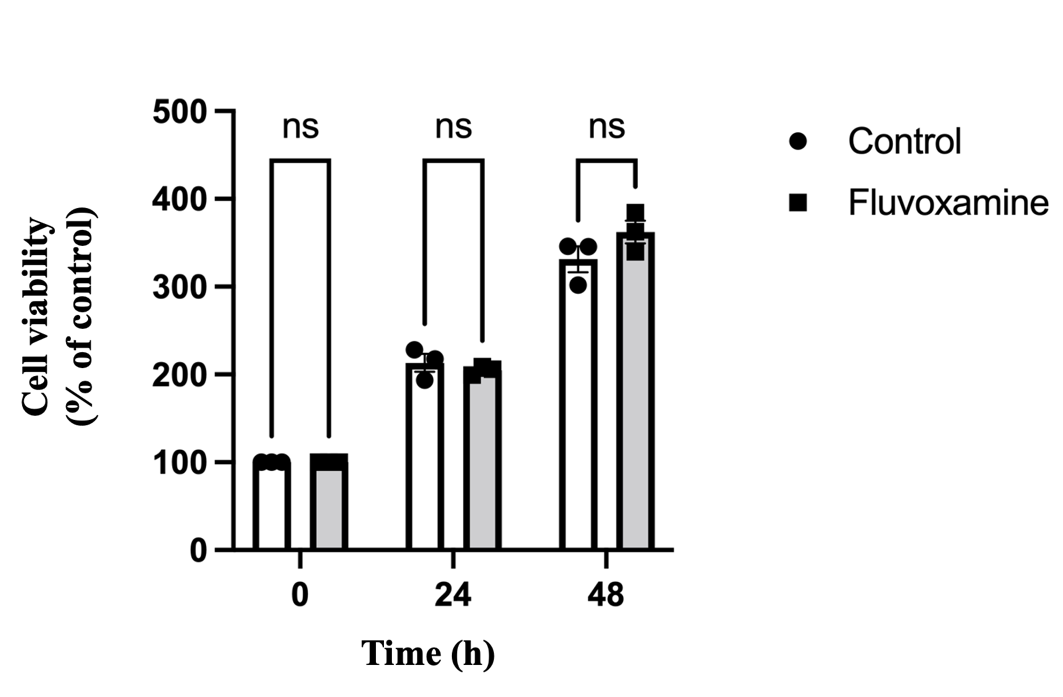
**

**Supplementary Fig. 1: Effect of Fluvoxamine on NSC34 cytotoxicity.** The CCK-8 assay was conducted to assess cell viability following Fluvoxamine treatment at various time intervals.

**
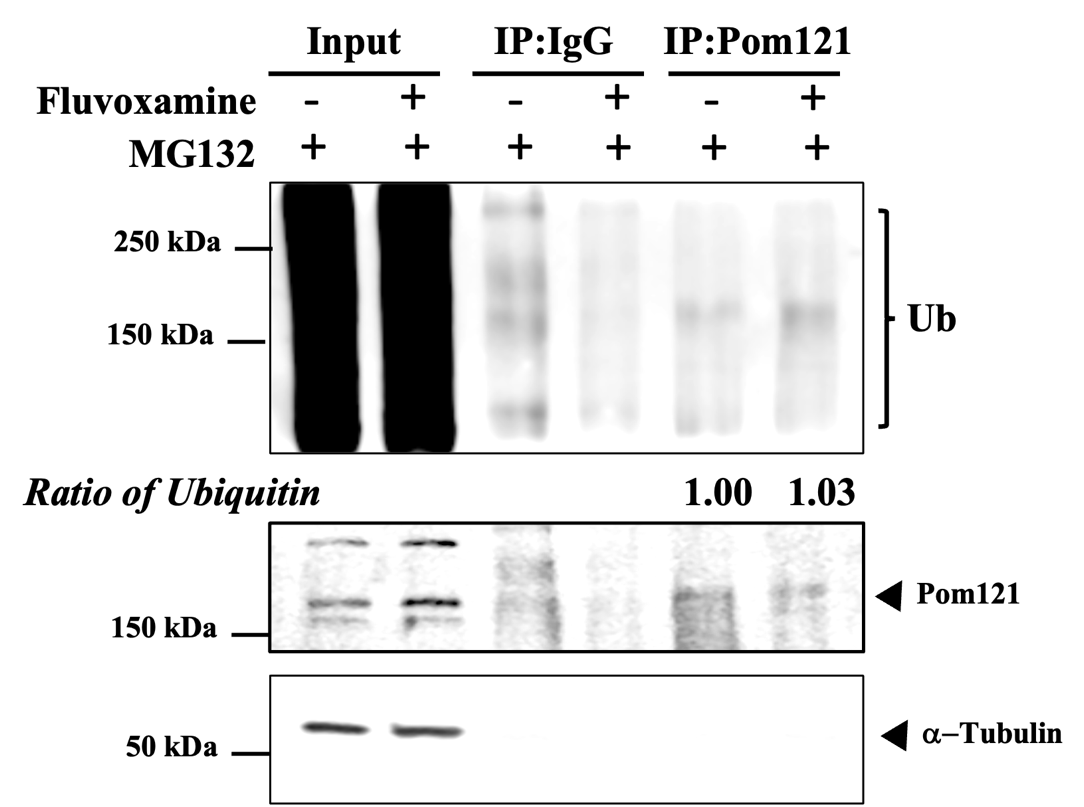
**

**Supplementary Fig. 2: Fluvoxamine induces the protein expression of Pom121 through a non-ubiquitin proteasome pathway.** An immunoprecipitation assay was performed to investigate the ubiquitination of Pom121. The NSC34 cells were treated with 10 µg/mL of Fluvoxamine for 1 hour. Subsequently, treated cells were incubated with MG132 for 6 hours. Following this treatment, protein extraction and an immunoprecipitation assay were conducted using anti-Pom121 as the primary antibody.

**
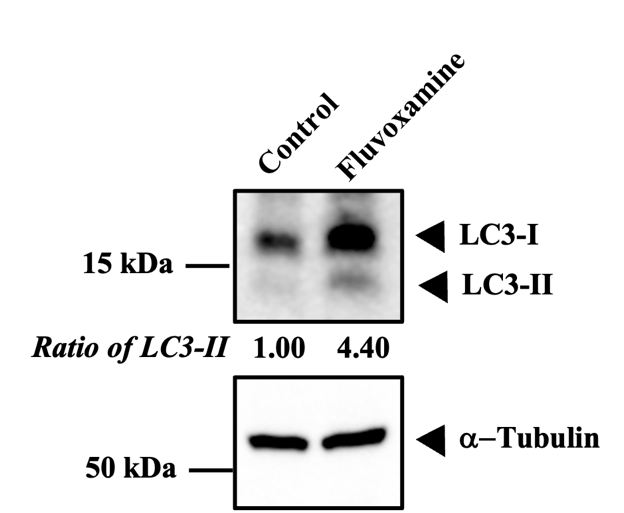
**

**Supplementary Fig. 3: LC3-II protein expression significantly increased under fluvoxamine treatment.** Protein extraction and western blotting were then performed. Anti-LC3 and anti-α-Tubulin were used as the primary antibodies. The results were quantified using ImageJ software.

**
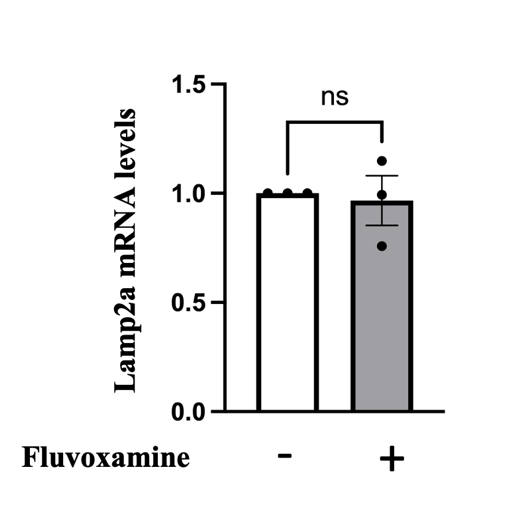
**

**Supplementary Fig. 4: Lamp2a mRNA exhibited no statistically significant difference under fluvoxamine treatment.** The transcription levels of Lamp2a were observed to increase upon treatment with fluvoxamine.

**Figure 1a shows the whole blot after cutting membrane at molecular weight 100 kDa, 75 kDa, 50 kDa, 37 kDa and 25 kDa for BiP (73 kDa), GFP (26 kDa), and Sigma-1R-GFP (50 kDa).**

**
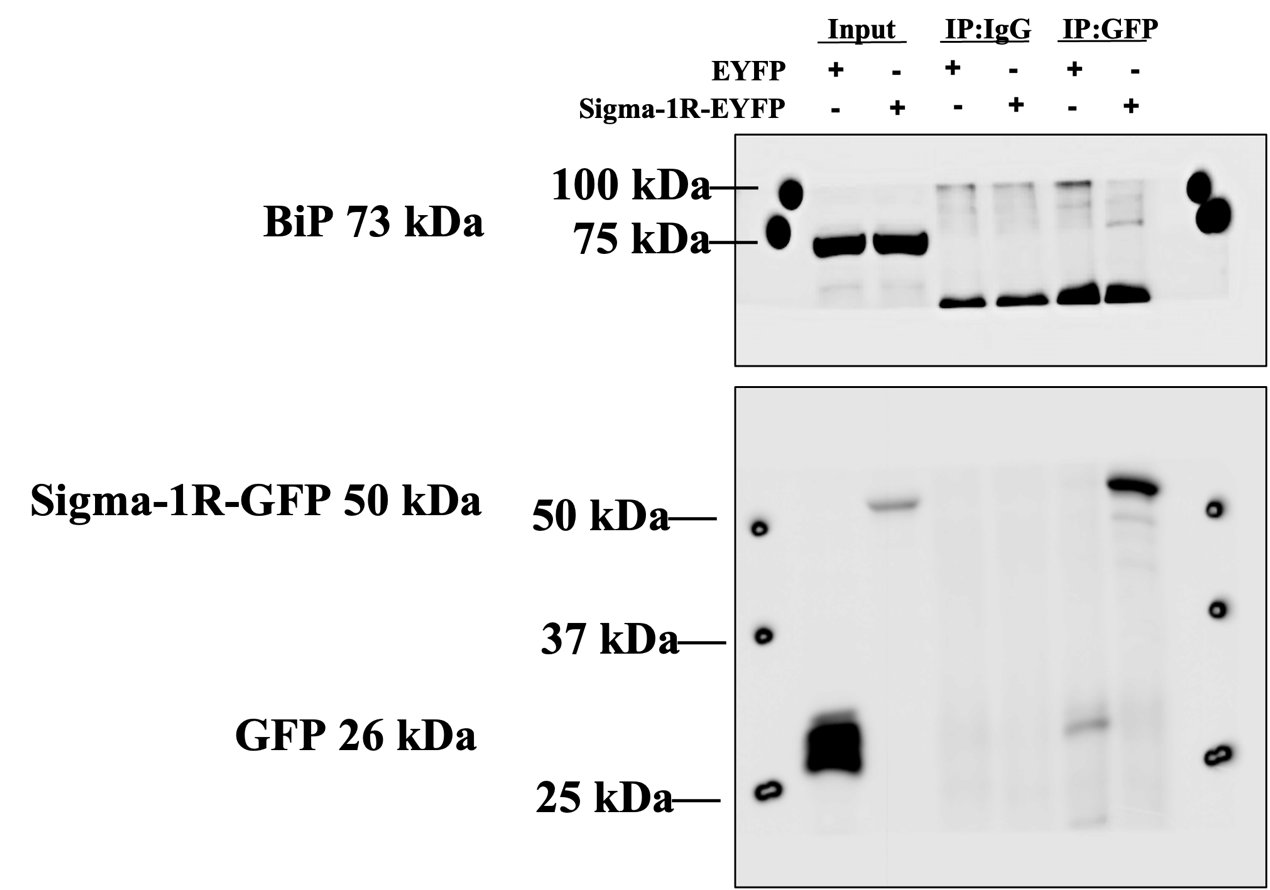
**

**Figure 1b shows the whole blot after cutting membrane at molecular weight 75 kDa, and 50 kDa for BiP (73 kDa), and Sigma-1R-GFP (50 kDa).**

**
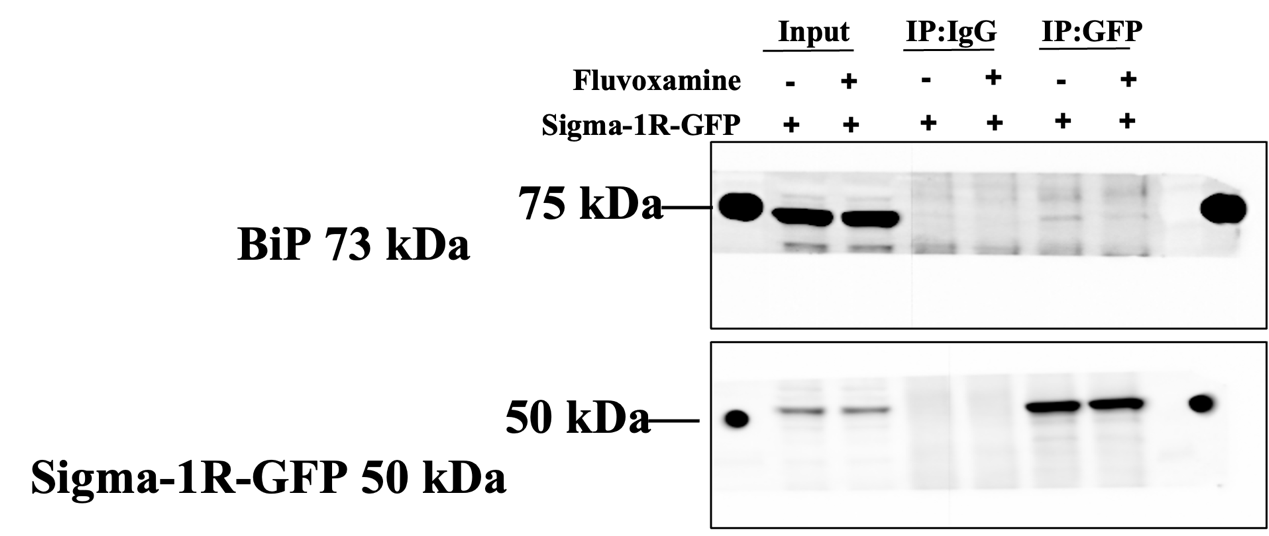
**

**Figure 3a shows the whole blot (4 times repeats) after cutting membrane at molecular weight 150 kDa, 250 kDa, 50 kDa and 37kDa for Pom121 (155 kDa), and Sigma-1R-GFP (50 kDa).**

**
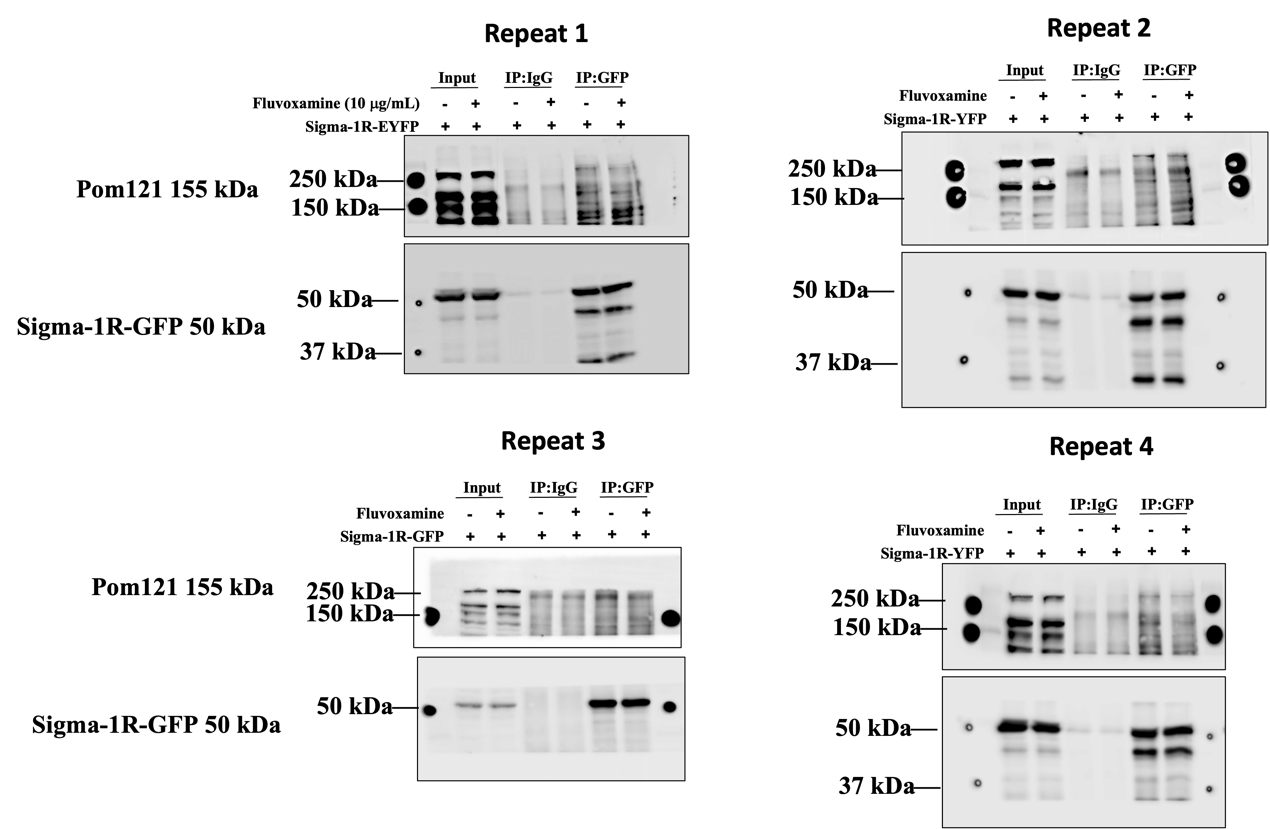
**

**Figure 3c-3e show the whole blot (3 times repeats) after cutting membrane at molecular weight 250 kDa, 150 kDa, 75 kDa, 50 kDa, 37kDa and 25 kDa for Pom121 (155 kDa), α-Tubulin (55 kDa), and EGFP-(G4C2)31 (26 kDa).**

**
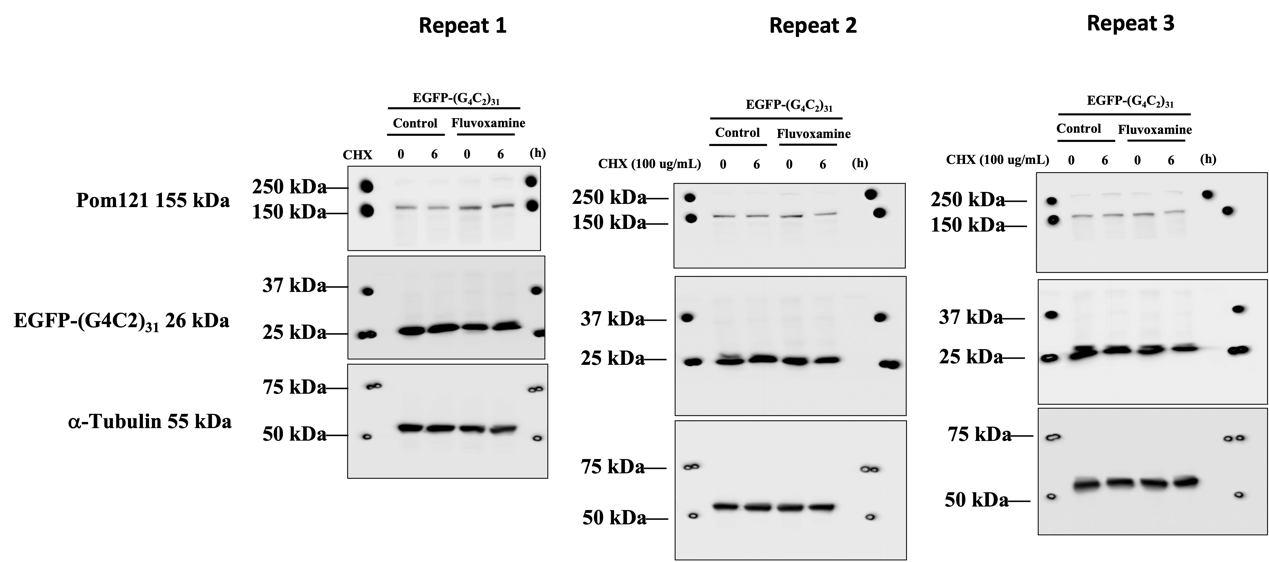
**

**Figure 4a-4c show the whole blot (4 times repeats) after cutting membrane at molecular weight 100 kDa, 75 kDa 37 kDa, and 25 kDa for TFEB (65-70 kDa), Importinβ1 (97 kDa), HDAC2 (60 kDa), β-Actin (43 kDa) and EGFP-(G4C2)31 (26 kDa).**

**
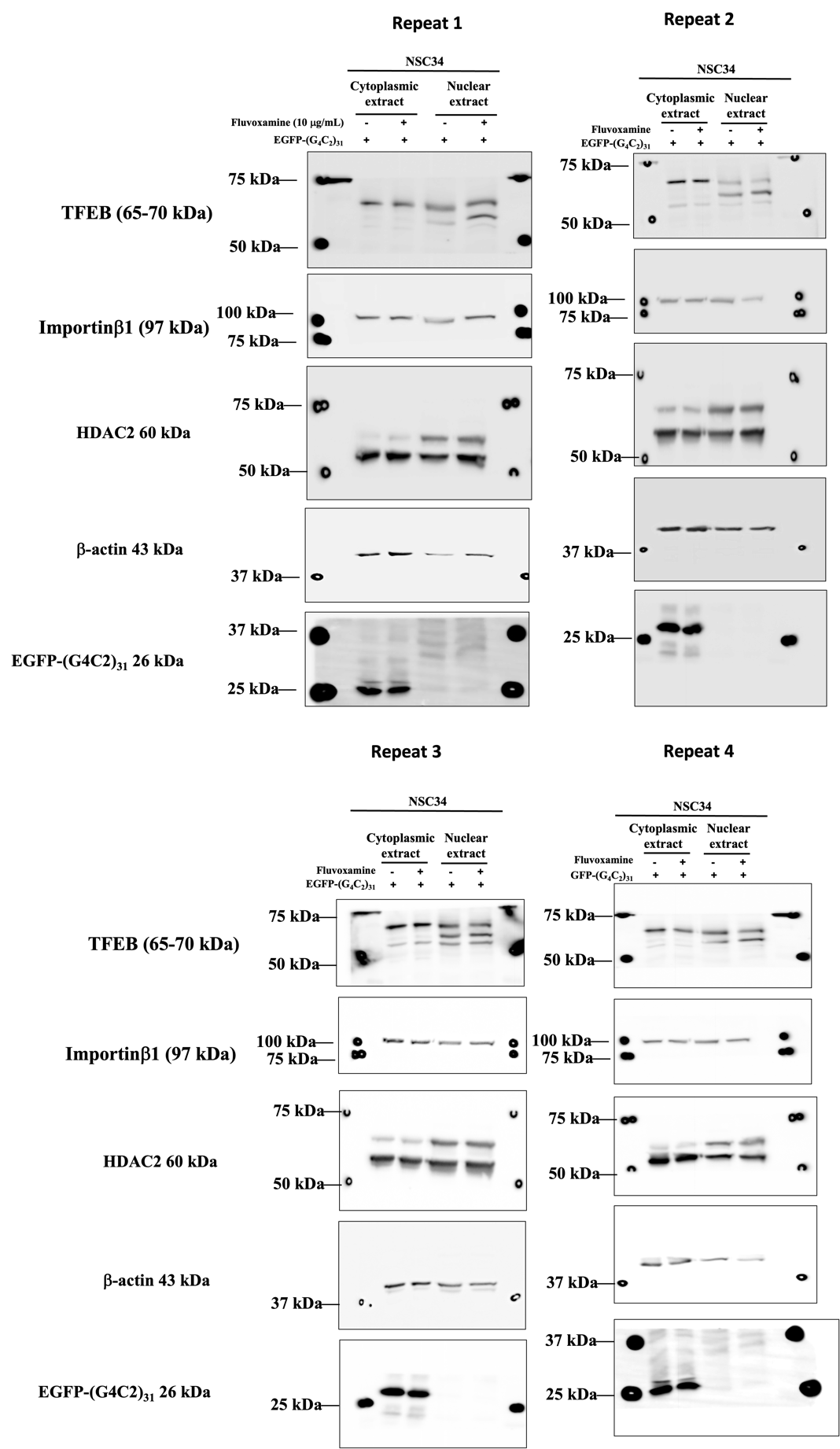
**

**Figure 4d-4e show the whole blot (5 times repeats) after cutting membrane at molecular weight 50 kDa, 25 kDa, and 15 kDa for LC3 (14-16 kDa), GFP (26 kDa), and Tubulin (55 kDa).**

**
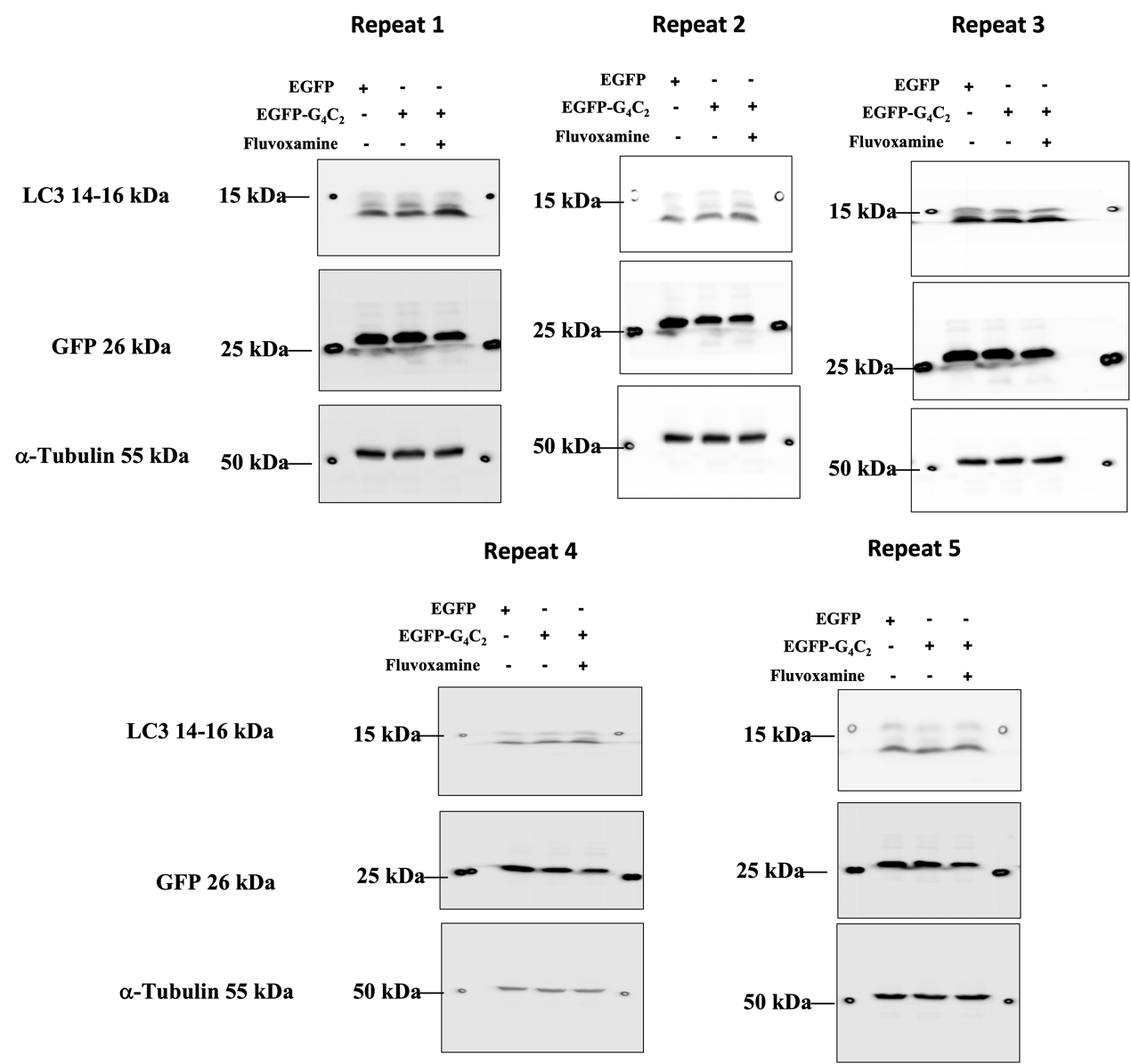
**
